# A Comprehensive Evaluation of Effects on Water-Level Deficits on Tomato Polyphenol Composition, Nutritional Quality and Antioxidant Capacity

**DOI:** 10.3390/antiox11081585

**Published:** 2022-08-16

**Authors:** Ning Jin, Li Jin, Shuya Wang, Xin Meng, Xianglan Ma, Xianxia He, Guobing Zhang, Shilei Luo, Jian Lyu, Jihua Yu

**Affiliations:** 1 College of Horticulture, Gansu Agricultural University, Lanzhou 730070, China; 2 State Key Laboratory of Arid Land Crop Science, Gansu Agricultural University, Lanzhou 730070, China

**Keywords:** water deficit, tomato, polyphenols, antioxidant capacity, nutritional quality, multivariate statistical analysis

## Abstract

Tomatoes have high nutritional value and abundant bioactive compounds. Moderate water deficit irrigation alters metabolic levels of fruits, improving composition and quality. We investigated the effects of water deficit (T1, T2, T3, and T4) treatments and adequate irrigation (CK) on tomato polyphenol composition, antioxidant capacity, and nutritional quality. Compared with CK, the total flavonoid content increased by 33.66% and 44.73% in T1 and T2, and total phenols increased by 57.64%, 72.22%, and 55.78% in T1, T2, and T3, respectively. The T2 treatment significantly enhanced antioxidant’ capacities (ABTS, HSRA, FRAP, and DPPH). There were multiple groups of significant or extremely significant positive correlations between polyphenol components and antioxidant activity. For polyphenols and antioxidant capacity, the classification models divided the treatments: CK and T4 and T1–T3. The contents of soluble solids, soluble protein, vitamin C, and soluble sugar of the treatment groups were higher than those of CK. The soluble sugar positively correlated with sugar–acid ratios. In the PCA-based model, T3 in the first quadrant indicated the best treatment in terms of nutritional quality. Overall, comprehensive rankings using principal component analysis (PCA) revealed T2 > T1 > T3 > T4 > CK. Therefore, the T2 treatment is a suitable for improving quality and antioxidant capacity. This study provides novel insights into improving water-use efficiency and quality in the context of water scarcity worldwide.

## 1. Introduction

Tomatoes (*Solanum lycopersicum* L.) belong to the Solanaceae family, with approximately 2800 species and an annual global production of over 161 million tons, making it one of the most-consumed vegetable commercial crops [1,2]. Tomatoes are often used as fresh fruit, cooked food, and processed products due to a range of human health benefits, such as antimicrobial [3], antioxidant [4], and protective effects against cardiovascular disease and cancer [5]. The demand for quality vegetables and fruits also increased gradually with rising living standards and importance placed on health. The taste and quality of tomato fruit are typically reflected by the composition and content of primary (i.e., soluble sugars, organic acids, and amino acids) and secondary metabolites (i.e., flavonoids and phenolic acids) [6]. Tomatoes have many pharmacological and nutritional properties of bioactive components, such as lycopene, polyphenolic compounds, pro-vitamin A, and vitamin C, which are considered natural antioxidant cancer preventing compounds [7]. The pathogenesis of many diseases, such as atherosclerosis, cardiovascular disease, neurodegenerative diseases, brain aging, and cancer, is closely linked to oxidative damage caused by reactive oxygen species (ROS) [8,9]. The concentration of polyphenolic compounds (i.e., flavonoids and phenolic acids), which are secondary metabolites in tomato fruit, strongly correlate with antioxidant capacity, and these compounds prevent oxidative damage to cells by reducing ROS levels [10,11]. The antioxidant potential of polyphenols in vegetables and fruits is effective in reducing the adverse effects of ROS induced by athletes during strenuous exercise [12]. Vitamin C (also known as ascorbic acid), a micronutrient, is an essential antioxidant in human diet along with polyphenols [13]. Soluble sugars and organic acids, which account for more than 50% of the dry matter, are the main osmoregulatory substances in tomato fruit and play a pivotal role in flavor [14].

Drought, which affects nearly 40–60% of the world’s agricultural land, is regarded as a major environmental constraint on agricultural production [15]. Water scarcity is a major bottleneck that restricts sustainable agricultural development, particularly in arid and semi-arid regions. However, agricultural extension requires large amounts of irrigation water, with 80–85% of the total available water reportedly used for agriculture, and most on-farm irrigation systems are poorly managed, resulting in inefficient irrigation [16,17]. Various studies have proved that climate change could seriously affect the supply and demand for irrigation water owing to the continuous increase in temperature and atmospheric CO_2_ levels that changed rainfall patterns and their distribution in recent years [18,19]. Innovative water-saving irrigation methods, such as deficit irrigation, were developed to address water scarcity and to improve water-use efficiency [20]. It is generally believed that water deficits will have a negative impact on yield and a positive impact on quality by increasing primary or secondary metabolites. However, the positive or negative response of fruits to water deficit strongly depends on genotypes, fruit development stage, stress intensity and duration, and the interaction between stress factors [21].

Fruit polyphenol composition and nutritional quality are affected and regulated by many factors, including variety, maturity, cultivation management, and environmental stress [22]. The improper use of the water-deficit approach can seriously affect the processes of various physiological and biochemical reactions in plants, such as relative leaf water content, photosynthetic fluorescence parameters, stomatal response, osmotic regulation, reactive oxygen species, and antioxidant response [23,24]. Ultimately, inappropriate water deficits can adversely affect crop development and productivity throughout the growing season [25,26]. However, no period or degree of water deficit adversely affects the quality of the fruit. An appropriate water deficit balances the contradiction between fruit yield and quality. For example, a short-term water deficit in the later stages of fruit growth improves the quality of nectarine fruit and, thus, increases the likelihood of consumer acceptance of fruit with lower yield losses [27]. Regulated deficit irrigation from fruits set to harvest decreased grape-berry organic acid content and increased soluble sugar and anthocyanin contents [28]. In addition, other studies observed that water deficit irrigation can effectively increase the total phenolic and proanthocyanidin contents of Cabernet Sauvignon grapes during ripening [29].

The proper use of water deficit irrigation has important practical implications for effectively improving water use efficiency and fruit quality in the context of global water shortages. Tomatoes are one of the main vegetables beneficial to human nutrition and health. To completely exploit innovative irrigation management technology, we need to improve our understanding of how water-deficit irrigation affects the quality of tomato fruits during development and evaluate the extent to which the quality of tomato fruits is affected. Multivariate statistical analyses have been successfully used for scientific classification and ranking in agricultural research [30,31]. Consequently, this study considered tomato fruits (cv. ‘Micro-Tom’) to evaluate the differences in polyphenol content, antioxidant activity (free radical scavenging capacity assessed according to ABTS, HSRA, FRAP and DPPH), and nutritional quality from mature green stages to red ripening under CK (normal water) and four water-deficit irrigation conditions. In this study, multivariate statistical analysis (i.e., a classification model based on principal component analysis and cluster analysis) was used to explore the effects of different water-shortage frequencies on tomato fruit quality and to conduct scientific classification, with the aim of providing a basis for subsequent research.

## 2. Materials and Methods

### 2.1. Plant Materials and Growth Conditions

Tomato seeds (*Solanum lycopersicum* L. cv. ‘Micro-Tom’) were purchased from PanAmerican Seed. Seeds were surface sterilized in 4% (*v*/*v*) sodium hypochlorite for 15 min and then rinsed several times with sterile distilled water [32]. Seeds were sown in 9 cm Petri dishes (approximately 20 seeds per dish) with the bottom covered with moist filter paper. Petri dishes were wrapped in Parafilm and placed in an artificial climate chamber (RDN-400E-4; Ningbo Dongnan Instrument Co. Ltd., Zhejiang, China) at 28 °C in the dark for germination. When a 0.3 cm radicle emerged, the seedlings were transplanted into 50-hole cavity trays for seedling development. When the seedlings reached three true leaves, they were transplanted into plastic pots (size: 7 × 7 × 8 cm) filled with a 2:1:1 (*v*/*v*/*v*) mixture of grass charcoal, vermiculite, and perlite substrate. Tomato plants were incubated under controlled climatic conditions (16/8 h light/dark cycle, 28/18 °C day/night cycle, 50% relative humidity, 300 μmol m^−2^ s^−1^ photosynthetic photon flux density).

### 2.2. Experimental Desizgn

Five treatments were set up with three replicates of 10 pots per treatment. Prior to the start of the water deficit treatment, all pots were immersed in water until they were fully saturated (approximately 3 cm depth for 12 h); the excess water was then drained and weighed (at this point, the water content of the substrate was the maximum field moisture capacity) [33]. Fruits from the same flowering period were marked. The water deficit was applied for 20 days after flowering (DAF) when the tomato fruits were in the mature green stage. The experimental treatments were as follows: (i) CK: fully irrigated treatment, which is 90% of the field’s moisture capacity; (ii) T1: the substrate moisture content is 80% of the field moisture capacity; (ⅲ) T2: the substrate water content is 65% of the field’s moisture capacity; (v) T3: the substrate moisture content is 55% of the field moisture capacity; (ⅳ) T4: the substrate moisture content is 45% of the field moisture capacity. The substrate’s moisture content was controlled by weight [34]. All indicators were determined after water-deficit treatments during red ripening. Tomato fruits of uniform size were collected, frozen in liquid nitrogen, and then stored at −70 °C for the determination of relevant indicators.

### 2.3. Determination Indices and Methods

#### 2.3.1. Determination of Polyphenols

For determination of the four flavonoids (rutin, quercetin, naringenin, and kaempferol) and 12 phenols (protocatechuic acid, p-hydroxybenzoic acid, chlorogenic acid, gallic acid, p-coumaric acid, ferulic acid, benzoic acid, cinnamic acid, gentisic acid, caffeic acid, cynarin, and sinapic acid), 0.1 g of tomato cold lyophilized powder was weighed, and 2 mL of methanol was placed in a 5 mL centrifuge tube and extracted at 25 °C for 1 h. The tubes were centrifuged at 4 °C for 10 min at 8000 rpm, and the supernatant was filtered through a 0.22 µm organic phase filter membrane and assayed. The supernatant samples were analyzed by high-performance liquid chromatography (HPLC) using a symmetrical C18 column (250 mm × 4.6 mm, 5 µm, Waters Corp., Milford, MA, United States). The flow rate was 1.1 mL min^−1^; injection volume was 10 µL; mobile phase: methanol (A), 1% (*v*/*v*) acetic acid (B); and column temperature was controlled at 30 °C. Gradient elution was performed. The compounds were detected at 240, 280, and 322 nm. The compounds were identified based on the retention times of the standards (YuanYe Biotechnology Co., Ltd. Shanghai, China) and quantified using a standard curve. Each treatment consisted of three biological replicates, with each replicate containing five tomato fruits.

#### 2.3.2. Determination of Antioxidant Parameters

The total antioxidant capacity of tomato fruits was determined according to the manufacturer’s instructions (Sino Best Biological Technology Co., Ltd., Shanghai, China), including 2,2′-azino-bis(3-ethylbenzothiazoline-6-sulfonic acid) (ABTS) radical scavenging activity, hydroxyl radical scavenging (HSRA), ferric-reducing antioxidant power (FRAP), and 2,2-diphenyl-1-picrylhydrazyl (DPPH) radical scavenging activity. Absorbance at 734, 536, 593, and 515 nm was measured using a UV-1800 visible spectrophotometer (SHIMADZU, Japan) according to the manufacturer’s protocol. The units of FRAP were U g^−1^ FW, and the units of ABTS, HSRA, and DPPH were all in %. The antioxidant capacity of the tomatoes was quantified based on the change in absorbance, and vitamin C was used as a positive control.

#### 2.3.3. Determination of Nutritional and Safety Qualities

Soluble solids were measured using a PAL-1 refractometer (Atago Co. Ltd., Tokyo, Japan). The Coomassie Brilliant Blue method was used to estimate soluble protein levels [35] and the 2,6-dichloroindophenol staining method was used to determine the vitamin C content [36]. The soluble sugar content was determined using the anthrone–sulfuric acid method [37]. The titratable acid content of the tomato fruit was determined following the method described by Wang et al. [38] using 0.1 mol L^−1^ NaOH (containing two drops of 1% phenolphthalein), pale pink in color, as an indicator. The nitrate content was determined using the salicylic acid–sulfuric acid method [39].

### 2.4. Statistical Analysis

The data were analyzed by one-way analysis of variance (ANOVA) using SPSS software (version 22.0; SPSS Institute Inc., Chicago, IL, USA), and the significant differences were compared by Duncan’s multiple range test (*p* < 0.05). Correlation analysis, principal component analysis (PCA), heat map generation, and hierarchical cluster analysis (HCA) were performed using Origin 2021 (Origin, Inc., San Francisco, CA, USA). Results are presented as mean ± standard error (SE).

## 3. Results

### 3.1. Effect of Different Levels of Water Deficit on Polyphenol Content and Antioxidant Parameters in Tomato Fruit

#### 3.1.1. Variance Analysis

The four water-deficit treatment groups increased the content of flavonoid compounds (rutin, quercetin, naringenin, and kaempferol) in tomato fruit to different degrees compared with CK. The T1, T2, T3, and T4 treatments significantly increased the rutin content of tomato fruits by 2.31, 2.68, 2.10, and 2.06, respectively, compared to CK, with the largest increase in the T2 treatment. Compared to CK, quercetin’s content increased by 13.13%, 20.75%, and 6.60% in the T1, T2, and T3 treatments, respectively, and decreased by 21.15% in the T4 treatment (Table 1). Similarly, compared to CK, the different water deficit treatment groups also increased by varying the degrees of the content of phenolic acid components (protocatechuic acid, p-hydroxybenzoic acid, chlorogenic acid, gallic acid, p-coumaric acid, ferulic acid, benzoic acid, cinnamic acid, gentisic acid, caffeic acid, cynarin, and sinapic acid) of tomato fruit. Protocatechuic acid, chlorogenic acid, gallic acid, and gentisic acid were markedly increased in T1, T2, and T3 treatments compared to CK. The benzoic acid content significantly increased by 141.29% and 157.69% with T2 and T3 treatments, respectively, and the cinnamic acid content significantly increased by 126.92% with the T2 treatment compared to CK.

**Figure 1:**
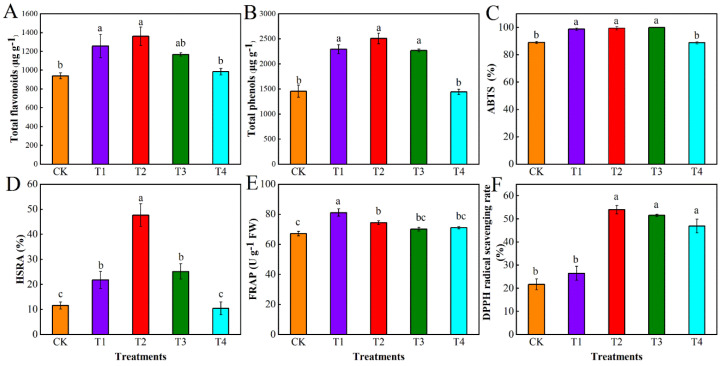
Effect of different levels of water deficit treatments on total flavonoids (**A**), total phenols (**B**), ABTS (**C**), HSRA (**D**), FRAP (**E**) and DPPH (**F**) of tomato fruits. ABTS: 2,2′-azino-bis (3-ethylbenzothiazoline-6-sulfonic acid; HSRA: hydroxyl radicals scavenging; FRAP: ferric-reducing antioxidant power; DPPH: 2,2-diphenyl-1-picrylhydrazyl radical scavenging activity. The abbreviations CK, T1, T2, T3, and T4 are defined in the footnote of Table 1. The data are presented as mean ± SE of three biological replicates. Different lowercase letters indicate significant differences by Duncan’s multiple range tests (*p* < 0.05).

Compared with CK, the total flavonoid content significantly increased by 33.66% and 44.73% in T1 and T2 treatments, respectively (Figure 1A); total phenols significantly increased by 57.64%, 72.22%, and 55.78% in T1, T2, and T3 treatments, respectively (Figure 1B). However, the total flavonoid and total phenolic contents of the T4 treatment were not significantly different from those of CK and even decreased. The impact of water deficits on the oxygen resistance of tomato fruits was assessed using four parameters: ABTS, HSRA, FRAP, and DPPH. ABTS and HSRA were significantly increased in the T1, T2, and T3 treatments compared with CK (Figure 1C,D). HSRA in the T2 treatment was 4.11-times higher than that in CK, which was the largest increase. FRAP was notably higher in T1 and T2 treatments by 20.75% and 10.78%, respectively, compared to CK (Figure 1E). DPPH free-radical scavenging significantly increased by 148.93%, 137.34%, and 116.07% in T2, T3, and T4 treatments, respectively, compared to CK (Figure 1F). These results imply that suitable water deficit treatments could promote secondary metabolism and significantly increase the content of total flavonoids and phenols, which in turn effectively improves the antioxidant properties of tomato fruits.

#### 3.1.2. Correlation Analysis

**Figure 2:**
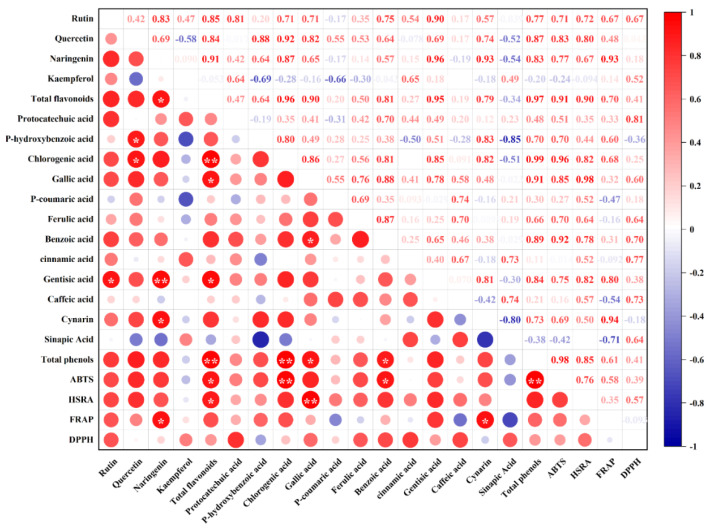
Pearson’s correlation analysis of polyphenols and antioxidant activity in tomato fruit under different levels of water deficit. ABTS, HSRA, FRAP, and DPPH are as defined in the legend of Figure 1. The abbreviations CK, T1, T2, T3, and T4 are defined in the footnote of Table 1. * and ** represent significant correlations at the *p* < 0.05 and *p* < 0.01 levels, respectively (two-tailed).

Pearson’s correlation analysis is regularly used to reveal the degree of correlation between the two parameters. As depicted in Figure 2, there were several sets of significant (*p* < 0.05) or highly significant (*p* < 0.01) positive correlations between the flavonoids and phenolic acids. A highly significant positive correlation was found between the total flavonoid content and chlorogenic acid content (r = 0.96). A highly significant positive correlation was also observed between naringenin and gentisic acid (r = 0.96). There were strong correlations between total phenols and total flavonoids and chlorogenic acid, with correlation coefficients up to 0.97 and 0.99, respectively. Similarly, we observed multiple significant or highly significant positive correlations between polyphenol levels and antioxidant activity. The FRAP was significantly positively correlated with naringenin (r = 0.93) and cynarin (r = 0.94). There were also significant positive correlations between ABTS (r = 0.91), HSRA (r = 0.90), and total flavonoids. HSRA and gallic acid (r = 0.98) were highly significantly correlated. ABTS, total phenols (r = 0.98) and chlorogenic acid (r = 0.96) were also significantly positively correlated.

#### 3.1.3. Principal Component Analysis and Cluster Analysis

**Figure 3:**
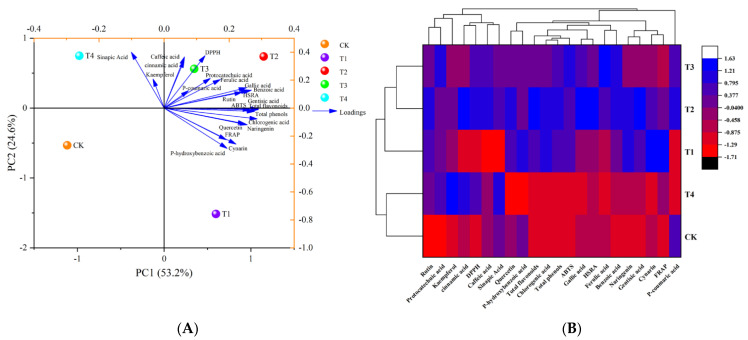
Principal component analysis (**A**) and cluster analysis (**B**) of polyphenols and antioxidant activity in tomato fruits under different levels of water deficit. ABTS, HSRA, FRAP, and DPPH are defined in Figure 1’s legend. The abbreviations CK, T1, T2, T3, and T4 are defined in the footnote of Table 1.

The classification model for polyphenols and antioxidant parameters of tomato fruit under different water deficit conditions based on principal component analysis is shown in Figure 3A, with the first and second principal components capturing most of the variation in the different water deficit treatments. The sum of the first two principal components for the water deficit treatments reached 77.8%, of which PC1 and PC2 explained 53.2% and 24.6% of the total variance, respectively. Furthermore, the loading plots demonstrated that total polyphenols and total phenols had strong first principal component loadings, and DPPH, sinapic acid, and caffeic acid had strong second principal component loadings and were utilized as proxy factors for tomato polyphenols and antioxidant activity in response to different levels of water deficit treatment. The CK, T4, T1, T2, and T3 treatments produced a visible separation based on PC1, while the T1, T2, and T3 treatments produced visible separation based on PC2. This classification result was corroborated by the results of the cluster analysis. The classification model based on cluster analysis likewise classified the five treatments into two broad categories: one for the CK and T4 treatments and the other for the T1, T2, and T3 treatments (Figure 3B).

### 3.2. Effects of Different Levels of Water Deficit on Nutritional Quality in Tomato Fruit

#### 3.2.1. Variance Analysis

**Figure 4:**
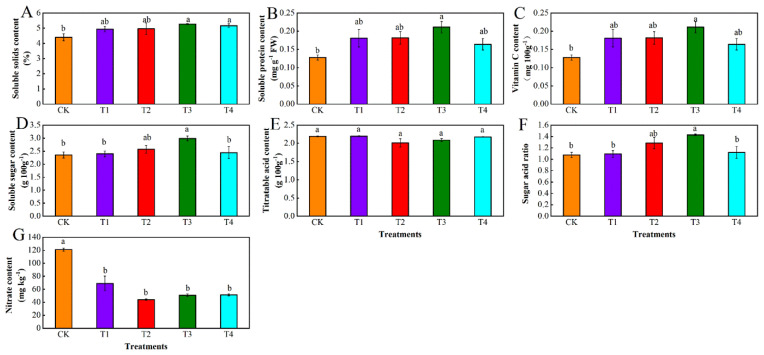
Effect of different levels of water deficit on the content of soluble solids (**A**), soluble protein (**B**), vitamin C (**C**), soluble sugar (**D**), titratable acid (**E**), sugar-to-acid ratio (**F**), and nitrate (**G**) in tomato fruit. The abbreviations CK, T1, T2, T3, and T4 are defined in the footnote of Table 1. The data are presented as mean ± SE of three biological replicates. Different lowercase letters indicate significant differences by Duncan’s multiple range tests (*p* < 0.05).

The soluble solids, soluble protein, vitamin C, and soluble sugar contents of tomato fruit treated with varying degrees of water deficit were higher than those of CK (Figure 4A–D). Of the four water deficit treatments, only soluble solids, soluble protein, vitamin C, and soluble sugars were significantly higher in T3 than in CK. Specifically, the soluble solids, soluble protein, vitamin C, and soluble sugar contents increased by 12.89%, 42.19%, 5.91%, and 9.26%, respectively, in the T2 treatment compared to CK. The contents of soluble solids, soluble protein, vitamin C, and soluble sugar significantly increased by 19.70%, 64.84%, 21.85%, and 26.93%, respectively, in the T3 treatment compared to CK. However, the water-deficit treatment did not have a statistically significant impact on the titratable butyric acid content of the tomato fruit (Figure 4E). The sugar-to-acid ratio increased by 19.53% in the T2 treatment compared to that in the CK treatment; the difference was insignificant. The sugar-to-acid ratio significantly increased by 32.93% in the T3 treatment compared to that in the CK treatment (Figure 4F). All four water-deficit treatments (T1–T4) had markedly lower nitrate contents than CK, with reductions of 42.91%, 63.46%, 58.02%, and 57.42%, respectively (Figure 4G).

#### 3.2.2. Correlation Analysis

**Figure 5:**
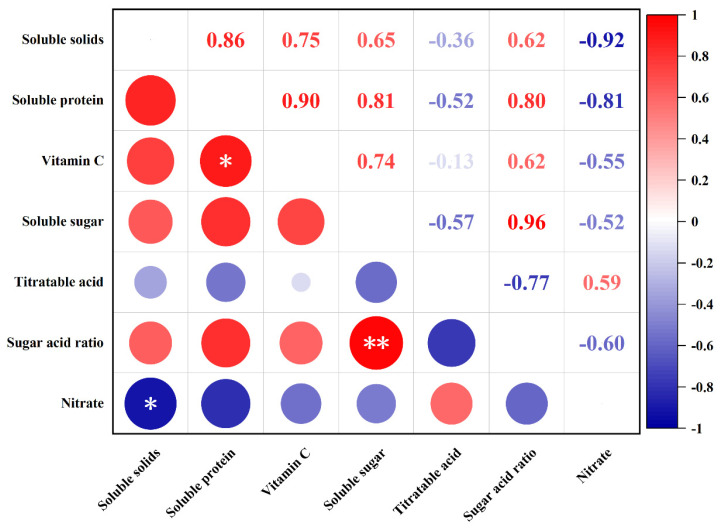
Pearson correlation analysis of nutritional quality under different levels of water deficit. The abbreviations CK, T1, T2, T3, and T4 are defined in the footnote of Table 1. * and ** represent significant correlations at the *p* < 0.05 and *p* < 0.01 levels, respectively (two-tailed).

The correlation between the nutritional quality of tomato fruits under water-deficit conditions is shown in Figure 5. Soluble sugars (r = 0.65), vitamin C (r = 0.81), and soluble protein (r = 0.74) positively correlated with soluble solids, except for nitrate (r = −0.92), which significantly and negatively correlated with soluble solids. Nitrate and soluble protein levels (r = −0.81) negatively correlated with each other. Additionally, vitamin C exhibited a significant positive correlation with soluble proteins (r = 0.90). There was a significant positive correlation between soluble sugars and the sugar–acid ratio (r = 0.96). Vitamin C and soluble sugar levels (r = 0.74) also showed a high positive correlation.

#### 3.2.3. Principal Component Analysis and Cluster Analysis

**Figure 6:**
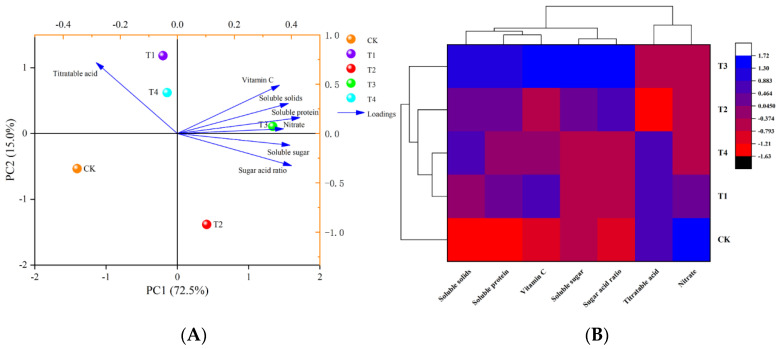
Principal component analysis (**A**) and cluster analysis (**B**) of the nutritional quality of tomato fruit under different levels of water deficit. The abbreviations CK, T1, T2, T3, and T4 are defined in the footnote of Table 1.

Figure 6A shows a classification model for the nutritional quality of tomato fruits under different water deficit conditions based on principal component analysis to generate a more effective visual representation. It can be observed that two principal components explained 87.5% of the total variance, with PC1 and PC2 accounting for 72.5% and 15.0%, respectively. Additionally, the loading plots revealed that the soluble protein and sugar-to-acid ratio were the main contributors to the first principal component, and titratable acid and vitamin C were the main contributors to the second principal component. Therefore, they were used as proxy factors for the quality of tomatoes in response to different levels of water-deficit treatments. Meanwhile, the CK and T1, T2, T3, and T4 treatment groups generated a clear separation based on PC1 and PC2. The T1 and T4 treatment groups and the T2 and T3 treatment groups generated clear separations based on PC1. Both T1 and T4 treatments were located in the second quadrant, indicating that they had more similar influences on tomato quality. CK was located in the third quadrant, T3 in the first quadrant, and T2 in the fourth quadrant. The classification model based on cluster analysis classified the five treatments into two categories: CK and T1, T2, T3, and T4 (Figure 6B). Of these, the T1 and T4 treatments were more similar in the T1–T4 category.

### 3.3. Evaluation of the Effects of Different Levels of Water-Deficit Treatments on Tomato Fruit Quality and Antioxidant Capacity Based on PCA

To better compare the effects of different levels of water deficits on tomato fruit quality and antioxidant capacity, we standardized 29 selected indicators using SPSS 22.0. The results of the principal component analysis extracted four principal components based on eigenvalues greater than one, with a cumulative variance contribution of 100% (Table 2). This indicates that the four principal components reacted to 100% of the information on the original 29 indicators. Based on the principal component loading values in Table S1, the highest variance contribution (50.30%) was obtained for principal component 1, which mainly combined the 22 indicators of rutin, quercetin, naringenin, total flavonoids, protocatechuic acid, p-hydroxybenzoic acid, chlorogenic acid, gallic acid, p-coumaric acid, ferulic acid, benzoic acid, gentisic acid, cynarin, total phenols, ABTS, HSRA, soluble solids, soluble protein, vitamin C, soluble sugar, sugar acid ratio, and nitrate information. The variance contribution of principal component 2 was 23.49%, with caffeic acid, sinapic acid, and DPPH loaded strongly onto it. The variance contribution of principal component 3 was 15.86%, which mainly combined information on kaempferol, FRAP, and titratable acid. The variance contribution of principal component 4 was 10.25%, and cinnamic acid had a greater loading.

To further compare the differences in tomato fruit quality and antioxidant capacity under different levels of water deficit, the software automatically calculated scores for each component based on the matrix and standardized data. The comprehensive scores were weighted by the variance contribution of each component and calculated as follows:

(1) $$Q_{i}=\overset{4}{\underset{m=1}{\sum }}P_{m}Z_{im}$$

where *Q* denotes the total score, *i* denotes the quality and antioxidant capacity of the *i*th of the five treatments, *m* denotes the *m* principal components, *P_m_* is the contribution of the *m* principal components to each other’s variance (Table 2), and *Z_im_* is the score of the *m* principal components of the quality and antioxidant capacity of the *i* treatments.

**Figure 7:**
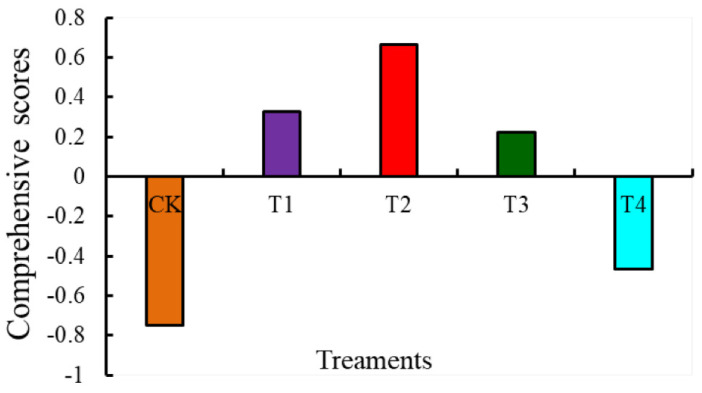
Comprehensive scores of tomato fruit quality and antioxidant capacity under different levels of water deficit. The abbreviations CK, T1, T2, T3, and T4 are defined in the footnote of Table 1.

Figure 7 shows the comprehensive scores for fruit quality and antioxidant capacity of tomatoes under different levels of water deficit. Using an overall score of 0 as a benchmark, positive values indicate improved quality and antioxidant capacity, higher values indicate improved quality and antioxidant capacity, and negative values indicate poorer quality and antioxidant capacity. The five treatments were ranked T2 > T1 > T3 > T4 > CK. Consequently, the T2 treatment was considered to be the most appropriate water-deficit treatment for improving tomato fruit quality and antioxidant capacity.

## 4. Discussion

In recent years, natural products, such as fruits and vegetables, attracted widespread attention from researchers and consumers as sources of phytochemicals. Phytochemicals in fruits and vegetables, owing to their biological properties, including antioxidant and free radical scavenging abilities, also appear to be partly responsible for human health-promoting effects, such as anticancer, anti-aging, hepatoprotective, antiviral activity, obesity, diabetes, and protection against cardiovascular disease [40]. Based on their structural and functional characteristics, phytochemicals can be classified into broad categories, such as polyphenols, nitrogen-containing compounds, terpenes, and organosulfur compounds [41]. Polyphenols are a widespread class of phytochemicals that have diverse biological functions in plants, such as responses to various biotic and abiotic stress factors. It is well known that in plants, polyphenols are mainly synthesized from phenylalanine produced by the shikimate pathway. Previous studies on grape berries by Ju et al. [42] showed that water-deficit irrigation treatments could increase phenylalanine levels in berries, thereby promoting polyphenol accumulation. Our study also showed that different levels of water deficit treatment promoted the accumulation of various components of polyphenols, including flavonoids and phenolic acids, to different degrees. According to Slimestad and Verheul [43], naringin is the main flavonoid found in tomatoes, followed by quercetin and kaempferol. However, in this study, the highest content of quercetin was observed in tomato fruits among the five treatments, which may be due to differences caused by different tomato varieties.

Phenolic acids, as small molecule metabolites, can be divided into two categories: hydroxycinnamic acids (caffeic acid, coumaric acid, ferulic acid, p-coumaric acid, chlorogenic acid, caffeic acid, etc.) and hydroxybenzoic acids (paraben, vanillic acid, syringic acid, gallic acid, protocatechuic acid, etc.) [44]. Chlorogenic acid is the main phenolic acid in tomato [43]. Similarly, in this study, the highest tomato phenolic acid content among the five treatments was chlorogenic acid. Most phenolic acid components were improved to some extent in the water-deficit treatment, which is consistent with our findings [45]. Remarkably, the content of various phenolic acids in the T2 treatment was significantly higher than that in the CK (protocatechuic acid, chlorogenic acid, gallic acid, gentisic acid, benzoic acid, and cinnamic acid). Nevertheless, the content of some phenolic acid components decreased in the T4 treatment, which may be due to an excessive water deficit.

Polyphenols have been shown to have ideal chemical structures for scavenging free radicals and are the main plant bioactive compounds with antioxidant activity [46]. The health value of fruit and vegetable polyphenols may be mediated by their effects on intestinal microbiota [47]. The accumulation of free radicals can lead to tissue damage and organ aging; therefore, free radical scavenging abilities can reflect the antioxidant capacity of fruits to a certain extent [30]. Generally, the higher the free radical scavenging rate, the stronger the antioxidant capacity of the fruit, and the better the quality. In the present study, we used ABTS, HSRA, FRAP, and DPPH to measure the change in free radical scavenging ability of tomato fruits under differing water deficit treatments. Total phenolic acids and total flavonoids significantly increased in the T2 treatment and significantly improved the free radical scavenging capacity of tomato fruit. Studies on *Amaranthus* leafy vegetables also indicated that suitable drought stress increased total phenolic acid content, total flavonoid content, and total antioxidant capacity (DPPH and ABTS) [48]. Antioxidants of interest to researchers and consumers are primarily derived from natural products, such as vegetables and fruits. Therefore, a deeper understanding of the correlation between these polyphenolic compounds and their free radical scavenging abilities is needed. Pearson’s correlation analysis indicated a positive correlation between total phenolic acids, flavonoids, DPPH, and ABTS (indicating free radical scavenging ability) [22,49,50]. There were also multiple groups of significant or extremely significant positive correlations between ABTS, HSRA, FRAP, and DPPH, and the components of polyphenols in our study.

Importantly, in the PCA-based classification model, the CK, T4, T1, T2, and T3 treatments were clearly separated by PC1, which is supported by the results of the HCA-based classification model. The results of PCA and HCA on polyphenols and antioxidant capacity suggested that water deficit (T1, T2, and T3) treatment could promote the accumulation of polyphenols and improve the antioxidant activity of fruits, especially in the T2 treatment. Thus, controlled water-deficit irrigation strategies can be used as abiotic stressors in signaling pathways involved in secondary metabolite synthesis, affecting fruit quality by inducing antioxidant metabolism and promoting the accumulation of bioactive compounds, which may bring benefits for consumers’ health.

Applying a moderate water deficit in the middle and late stages of fruit development optimizes fruit quality with small yield losses [51,52]. Water deficit increased soluble-sugar accumulation and decreased organic-acid content in both grape [28,45] and tomato crops [52]. However, Terry et al. [53] found that the water deficit increased sugar contents but did not affect acid content in strawberries, which is consistent with our results. The absolute amount and balance between sugars and acids determine the taste of the fruit and contribute to its overall flavor [54]. In our study, Pearson’s correlation analysis also showed that the soluble sugar content and sugar-to-acid ratio had a very significant positive correlation. In several tomato genotypes, moderate water deficits have been shown to increase the sugar-to-acid ratio of fruit [52,55]. Likewise, the sugar-to-acid ratio of the T3 treatment was significantly higher than that of CK by 32.93% in our study. The initial physiological response of plants to water deficit conditions is osmoregulation, which involves the accumulation or de novo synthesis of intracellular solutes, resulting in changes in cell osmotic potential [56].

Soluble proteins, as major osmotic regulators, have been shown to accumulate under water deficits [57,58]. Similarly, soluble protein accumulated significantly in the tomatoes with the T3 treatment. Under greenhouse cultivation conditions, soluble sugars, organic acids, vitamin C, and lycopene tend to decrease at high irrigation levels [59]. Interestingly, Massot et al. [60] suggested that vitamin C content may decrease during cell division and expansion due to dilution effects and then increase with increasing hexose content during ripening. Furthermore, studies have shown a strong correlation between fruit soluble sugars and vitamin C content [61]. In this study, vitamin C and soluble sugar contents in tomato fruit increased under suitable water deficit conditions, with high positive correlations between them. A PCA- and HCA-based classification model for nutritional quality suggested that water deficit (T1, T2, T3, and T4) treatments could promote tomato fruit’s nutritional quality, especially with the T3 treatment.

For polyphenol composition and antioxidant activity, the optimal water deficiency treatment was T2, while it was T3 for nutritional quality. Therefore, a suitable multi-indicator comprehensive evaluation model is needed to integrate the information of polyphenol composition, antioxidant activity, and nutritional quality to select the most beneficial water deficit treatments for tomato quality improvement. The choice of the model is directly related to the scientific rigor and accuracy of the evaluation results. PCA is a statistical analysis method that converts multiple indicators into several comprehensive indicators and has become one of the main methods for the comprehensive quality evaluation of fruits, vegetables, and food [62,63]. Furthermore, these composite indicators, called principal components, should contain as much information as possible from original variables. The PCA method avoids bias caused by subjective factors, and the selected principal components should cover more than 80% of the total information in the original data [64]. PCA can be used not only for visual classification but also for comprehensive ranking. For example, Jin et al. used PCA to rank the polyphenols and amino acids of 69 cabbage varieties [65]. Zhou et al. used PCA to comprehensively evaluate 14 flavonoids in Daphne Genkwa [64]. Cao et al. comprehensively evaluated and screened tomato cold tolerance using PCA [66]. In the present study, the results of the comprehensive ranking using principal component analysis based on 29 selected raw indicators of tomato fruit polyphenols, antioxidant capacity, and nutritional quality were T2 > T1 > T3 > T4> CK.

## 5. Conclusions

In this study, the four water-deficiency treatment groups showed varying degrees of flavonoid and phenolic acid content in the tomato fruit. Compared to CK, total flavonoids significantly increased in T1 and T2 treatments, and total phenols significantly increased in T1, T2, and T3 treatments. There were multiple groups of significant or extremely significant positive correlations between the flavonoids and phenolic acids. Similarly, we observed multiple groups of significant or extremely significant positive correlations between polyphenol levels and antioxidant activity. The PCA-based classification model showed that the CK, T4, T1, T2, and T3 treatments produced distinct separations based on PC1. T1, T2, and T3 produced a clear separation based on PC2. This classification result was corroborated by HCA results. The HCA-based classification model also divides the five treatments into two categories: one for CK and T4 treatments and the other for T1, T2, and T3 treatments. Different degrees of water deficit affected the contents of flavonoids and phenolic acids and further improved the antioxidant capacity of tomato fruit, especially in the T2 treatment. The water-deficit treatment promoted the accumulation of soluble solids, soluble protein, vitamin C, and soluble sugar in tomato fruits. The soluble sugar and sugar–acid ratio (r = 0.96) showed a significant positive correlation. The PCA-based classification model showed that both T1 and T4 treatments fell into the second quadrant, indicating that their effects on tomato quality were similar. Furthermore, T3 fell into the first quadrant, indicating that the T3 treatment was more conducive to the improvement of tomato nutritional quality. Similarly, the HCA-based classification model divided the five treatments into CK and T1, T2, T3, and T4 treatments. Based on the 29 selected original indicators of tomato fruit polyphenols, antioxidant capacity, and nutritional quality, the ranking of the five treatments using PCA was T2 > T1 > T3 > T4 > CK. In conclusion, the T2 treatment is an appropriate and promising water-deficit irrigation strategy for improving tomato quality and enhancing its antioxidant capacity.

## Figures and Tables

**Table 1:** Effect of different levels of water deficit on the content of polyphenol components (μg g^−1^ DW) in tomato fruit.

Treaments		CK	T1	T2	T3	T4
Flavonoids	Rutin	160.65 ± 25.66 ^b^	371.63 ± 37.97 ^a^	429.93 ± 40.94 ^a^	337.22± 9.41 ^a^	331.47 ± 22.47 ^a^
	Quercetin	633.07 ± 18.24 ^ab^	716.21 ± 85.74 ^a^	764.43 ± 69.00 ^a^	674.87 ± 15.35 ^ab^	499.19 ± 41.65 ^b^
	Naringenin	144.66 ± 13.74 ^a^	166.06 ± 4.20 ^a^	163.39 ± 8.96 ^a^	152.52 ± 4.37 ^a^	150.09 ± 1.70 ^a^
	Kaempferol	1.53 ± 0.31 ^b^	2.33 ± 0.38 ^b^	2.58 ± 0.66 ^b^	2.16 ± 0.25 ^b^	3.89 ± 0.35 ^a^
Phenols	Protocatechuic acid	8.69 ± 0.19 ^b^	33.66 ± 4.34 ^a^	37.45 ± 3.76 ^a^	44.96 ± 6.04 ^a^	41.95 ± 4.72 ^a^
	P-hydroxybenzoic acid	18.95 ± 0.82 ^ab^	22.74 ± 1.28 ^a^	20.01 ± 3.05 ^a^	19.16 ± 1.65 ^ab^	13.78 ± 1.19 ^b^
	Chlorogenic acid	1195.52 ± 112.65 ^b^	1811.66 ± 60.22 ^a^	1871.26 ± 82.64 ^a^	1651.91 ± 58.10 ^a^	1087.77 ± 68.75 ^b^
	Gallic acid	31.25 ± 2.69 ^c^	50.08 ± 6.99 ^b^	88.64 ± 5.41 ^a^	63.83 ± 7.77 ^b^	28.09 ± 1.79 ^c^
	P-coumaric acid	0.52 ± 0.10 ^a^	0.33 ± 0.06 ^a^	0.54 ± 0.18 ^a^	0.50 ± 0.01 ^a^	0.31 ± 0.07 ^a^
	Ferulic acid	9.92 ± 0.24 ^a^	9.44 ± 3.12 ^a^	14.61 ± 4.82 ^a^	17.40 ± 1.04 ^a^	8.50 ± 1.72 ^a^
	Benzoic acid	165.74 ± 28.56 ^b^	302.62 ± 84.04 ^ab^	399.91 ± 43.28 ^a^	427.1 ± 28.53 ^a^	220.18 ± 23.42 ^b^
	Cinnamic acid	1.30 ± 0.22 ^c^	1.13 ± 0.04 ^c^	2.95 ± 0.70 ^a^	1.57 ± 0.31 ^bc^	2.54 ± 0.24 ^ab^
	Gentisic acid	16.87 ± 2.70 ^c^	53.32 ± 5.35 ^a^	62.23 ± 6.30 ^a^	33.63 ± 4.85 ^b^	30.86 ± 4.07 ^bc^
	Caffeic acid	0.53 ± 0.02 ^ab^	0.43 ± 0.02 ^b^	0.62 ± 0.02 ^a^	0.58 ± 0.09 ^ab^	0.53 ± 0.04 ^ab^
	Cynarin	3.92 ± 0.39 ^b^	7.15 ± 0.80 ^a^	5.76 ± 0.73 ^ab^	4.31 ± 0.83 ^b^	3.63 ± 0.60 ^b^
	Sinapic acid	2.07 ± 0.26 ^b^	1.54 ± 0.17 ^ab^	2.23 ± 0.40 ^ab^	2.15 ± 0.21 ^ab^	2.42 ± 0.08 ^a^

Note: Different lowercase letters in the same row of the table indicate significant differences between treatments (*p* < 0.05). Abbreviations: CK: fully irrigated treatment, which was 90% of the field moisture capacity; T1: substrate moisture content was 80% of the field moisture capacity; T2: substrate water content was 65% of the field moisture capacity; T3: substrate moisture content was 55% of the field moisture capacity; T4: substrate moisture content was 45% of the field’s moisture capacity.

**Table 2:** Eigenvalue and accumulative contribution rate of tomato fruit quality and antioxidant capacity under different levels of water deficit.

Component Number	Eigenvalues	% of Variance	Cumulative %
1	14.59	50.30	50.30
2	6.81	23.49	73.78
3	4.63	15.96	89.75
4	2.97	10.25	100.00

Extraction method: principal component analysis.

## Data Availability

All of the data are contained in the article and Supplementary Materials.

## Supplementary Materials

The following supporting information can be downloaded at: https://www.mdpi.com/article/10.3390/antiox11081585/s1, Table S1: Component matrix of tomato fruit quality and antioxidant capacity under different levels of water deficit.
