# Supplementary material for: A Comprehensive Evaluation of Effects on Water-Level Deficits on Tomato Polyphenol Composition, Nutritional Quality and Antioxidant Capacity
Source: Antioxidants (Basel). 2022 Aug 16;11(8):1585. doi: 10.3390/antiox11081585 (PMC9405155; doi:10.3390/antiox11081585)
Supplement: Supplementary file 1 [file antioxidants-11-01585-s001.zip › antioxidants-1859411-supplementary.pdf]

Table S1. Component matrix of tomato fruit quality and antioxidant capacity under different levels of water deficit.

Index	Component			
	1	2	3	4
Rutin	0.863	0.044	0.449	0.228
Quercetin	0.717	-0.519	-0.449	0.124
Naringenin	0.757	-0.513	0.329	0.236
Kaempferol	0.023	0.506	0.818	0.273
Total flavonoids	0.932	-0.293	0.019	0.212
Protocatechuic acid	0.677	0.439	0.558	-0.196
P-hydroxybenzoic acid	0.463	-0.821	-0.309	-0.125
Chlorogenic acid	0.896	-0.429	-0.110	0.018
Gallic acid	0.923	0.008	-0.305	0.236
P-coumaric acid	0.257	0.148	-0.949	0.110
Ferulic acid	0.730	0.349	-0.506	-0.299
Benzoic acid	0.964	0.151	-0.109	-0.191
Cinnamic acid	0.321	0.618	0.167	0.698
Gentisic acid	0.826	-0.306	0.253	0.401
Caffeic acid	0.367	0.704	-0.534	0.293
Cynarin	0.562	-0.789	0.208	0.132
Sinapic acid	-0.130	0.947	-0.075	0.285
Total phenols	0.957	-0.273	-0.098	-0.011
ABTS	0.940	-0.258	-0.096	-0.200
HSRA	0.866	-0.016	-0.269	0.422
FRAP	0.507	-0.667	0.537	0.098
DPPH	0.650	0.742	0.115	0.113
Soluble solids	0.601	0.493	0.547	-0.310
Soluble protein	0.856	0.185	0.212	-0.433
Vitamin C	0.599	0.004	0.275	-0.752
Soluble sugar	0.611	0.484	-0.239	-0.579
Titratable acid	-0.777	-0.396	0.371	-0.319
Sugar acid ratio	0.732	0.498	-0.308	-0.348
Nitrate	0.762	0.417	0.492	0.053

Extraction method: principal component analysis. 4 components extracted.
ABTS, HSRA, FRAP and DPPH are as defined in the Figure 1 legend.
